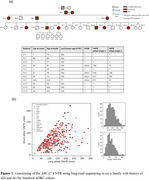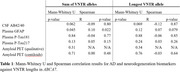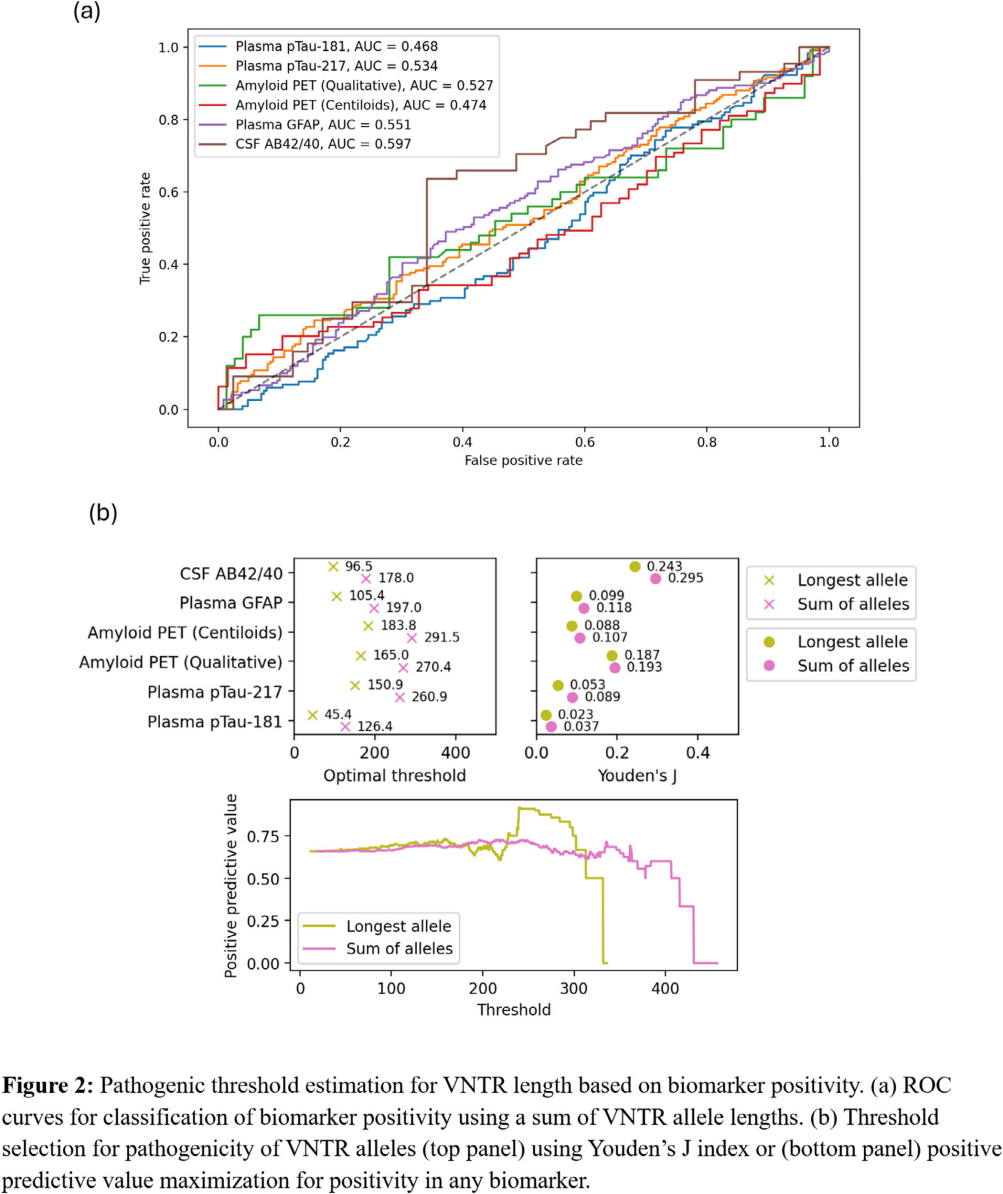# Biomarker‐informed characterization of an *ABCA7* tandem repeat expansion in a family with early‐onset Alzheimer’s Disease

**DOI:** 10.1002/alz70861_108977

**Published:** 2025-12-23

**Authors:** Andrés Peña‐Tauber, Lia Talozzi, Holden Orias, Elizabeth C. Mormino, Edward N. Wilson, Michael D. Greicius

**Affiliations:** ^1^ Stanford University, Stanford, CA USA

## Abstract

**Background:**

ATP‐binding cassette transporter A7 (*ABCA7*) has been genetically implicated in Alzheimer’s Disease (AD). A recent study by De Roeck et al. (2018) suggested a variable number tandem repeat (VNTR) in intron 18 of *ABCA7* is a driver of the genetic signal at this locus. Using long‐read sequencing (LRS), we sought to characterize this VNTR in a family with a strong history of AD and an atypical presentation (PMCID: PMC11709698), as well as in a larger dementia dataset.

**Method:**

We used Straglr‐v1.5.3 to genotype the VNTR in the family as well as 568 participants with mixed cognitive status in the Stanford AD Research Center (ADRC). Participants were assessed with various AD biomarkers. We tested VNTR lengths against each biomarker using Spearman correlation, Mann‐Whitney U test, and receiver operating characteristic (ROC) curves. We then determined optimal cutoffs to define a VNTR genotype as pathogenic using Youden’s J index and positive predictive value.

**Result:**

The index case had AD onset at 67 years and presented with amyloid‐PET negativity despite being plasma biomarker positive, tau‐PET positive, and moderately symptomatic. He carried two large VNTR alleles at 219 and 195 copies. Onset age in the case’s family appeared to be associated with the number of large VNTR alleles [Figure‐1]. In the ADRC dataset, a sum of VNTR allele lengths was associated with GFAP levels by Mann‐Whitney U (p = 0.045) and Spearman correlation (R = 0.10, *p* = 0.022), and suggestive associations were seen with CSF AB42/40 and plasma pTau‐217 [Table‐1]. Areas under the ROC curve for classification of biomarker positivity from VNTR length ranged from 0.468 to 0.597. Pathogenic cutoffs determined by Youden’s J ranged from 126.4 to 291.5 total VNTR units [Figure‐2]. Autopsy results on three family subjects showed end‐stage AD pathology and moderate‐to‐severe cerebral amyloid angiopathy (CAA).

**Conclusion:**

Expansion of this intronic VNTR in *ABCA7* appears to be associated with AD and possibly CAA. Our results are consistent with the pathogenic threshold of approximately 229 VNTR units determined by De Roeck et al. (2018). Validation in larger cohorts with LRS will be required to confirm the causality and mechanisms of this VNTR in AD.